# Adipose-derived mesenchymal stem cells' adipogenesis chemistry analyzed by FTIR and Raman metrics

**DOI:** 10.1016/j.jlr.2024.100573

**Published:** 2024-06-04

**Authors:** Karolina Augustyniak, Monika Lesniak, Hubert Latka, Maciej P. Golan, Jacek Z. Kubiak, Robert Zdanowski, Kamilla Malek

**Affiliations:** 1 Department of Chemical Physics, Faculty of Chemistry, Jagiellonian University in Krakow, Krakow, Poland; 2 Doctoral School of Exact and Natural Sciences, Jagiellonian University in Krakow, Krakow, Poland; 3 Laboratory of Molecular Oncology and Innovative Therapies, Military Institute of Medicine – National Research Institute, Warszawa, Poland; 4 Institute of Psychology, The Maria Grzegorzewska University, Warsaw, Poland; 5 Dynamics and Mechanics of Epithelia Group, Institute of Genetics and Development of Rennes (IGDR), Faculty of Medicine, University of Rennes, CNRS, UMR 6290, Rennes, France

**Keywords:** adipogenesis, mesenchymal stem cells, prediction of differentiation stage, regenerative medicine, spectroscopic molecular imaging

## Abstract

The full understanding of molecular mechanisms of cell differentiation requires a holistic view. Here we combine label-free FTIR and Raman hyperspectral imaging with data mining to detect the molecular cell composition enabling noninvasive monitoring of cell differentiation and identifying biochemical heterogeneity. Mouse adipose-derived mesenchymal stem cells (AD-MSCs) undergoing adipogenesis were followed by Raman and FT-IR imaging, Oil Red, and immunofluorescence. A workflow of the data analysis (IRRSmetrics4stem) was designed to identify spectral predictors of adipogenesis and test machine-learning (ML) methods (hierarchical clustering, PCA, PLSR) for the control of the AD-MSCs differentiation degree. IRRSmetrics4stem provided insights into the chemism of adipogenesis. With single-cell tracking, we established IRRS metrics for lipids, proteins, and DNA variations during AD-MSCs differentiation. The over 90% predictive efficiency of the selected ML methods proved the high sensitivity of the IRRS metrics. Importantly, the IRRS metrics unequivocally recognize a switch from proliferation to differentiation. This study introduced a new bioassay identifying molecular markers indicating molecular transformations and delivering rapid and machine learning-based monitoring of adipogenesis that can be relevant to other differentiation processes. Thus, we introduce a novel, rapid, machine learning-based bioassay to identify molecular markers of adipogenesis. It can be relevant to identification of differentiation-related molecular processes in other cell types, and beyond the cell differentiation including progression of different cellular pathophysiologies reconstituted in vitro.

Stem cells (SCs) are pluripotent cells that can both proliferate indefinitely and differentiate into numerous sorts of cells and tissues. These unique capacities allow for replacing damaged cells and tissues or participating in their repair or reconstruction (1, 2). Accordingly, they became the key tools of regenerative medicine and of many new therapies, allowing the treatment of even the most difficult cases without solution using traditional approaches (3, 4, 5, 6, 7). In particular, adult mesenchymal stem cells (MSCs) are of special significance in tissue engineering and regenerative medicine as they exhibit multi-lineage ability toward osteoblasts, chondrites, myoblasts, adipocytes, and neurons as well as participate in angiogenesis (2). The bone marrow and adipose tissues are the most well-known sources of MSCs. Obtaining sufficient MSCs from bone marrow for medical treatment is problematic due to its comparatively low percentage as well as the burden of medical intervention (8). As a result, there is a continuing sought for alternate, easily accessible, and high-yielding MSC procurement sources, with adipose tissue (adipose-derived mesenchymal stem cells – AD-MSCs) appearing to be the best suited (9, 10, 11).

There is a considerable risk of acquiring different forms of tumors (e.g., teratomas) if non-differentiated cells are introduced to the body in regenerative therapies. On the other hand, the administration of fully differentiated MSCs, for example, adipocytes, is associated with a higher risk of immune rejection. Therefore, striking the right balance of differentiating cells is essential for their eventual medical use (12). The detection of the expression of cell-type-specific markers is a critical step for identifying the initial population of stem cells and characterizing the level of differentiation. For this purpose, immunocytochemical methods, histological staining, flow cytometry, and reverse transcriptase polymerase chain reaction (RT-PCR) are commonly employed but a unique set of markers have not been proposed so far (13, 14, 15, 16). However, these tools do not provide precise information. The use of protein markers (mostly from the TNF- family of cancer necrotic factors) is one of the most fundamental methods of measuring cell development (17). Cell transplants, as well as histological and microscopic characterization, are necessary. All of them require the use of labels targeted to a specific biomarker and sophisticated, costly equipment, as well as the proper preparation of the sample. Multiple labels, appropriate for the successive stages of the researched mechanism, must be utilized to track such a complex process as cell differentiation (17). Therefore, to recognize the biochemical alternations of MSC differentiation and establish the trajectory of mature cell formation, especially at the early stage, novel molecular methods need to be developed (18, 19).

By being an in vitro and ex vivo non-invasive, non-label tool for clinical and biological purposes, Infrared (IR) and Raman (RS) microscopy garnered significant interest over the last decades. As there are no limits to the objects that can be investigated, multiple research initiatives have demonstrated so far more beneficial outcomes of IR and Raman strategies in the context of their pre/clinical usefulness for biological fluids (20), histopathology (21), cytology (22), microbiology (23), pharmacology (24), and biomarker exploration (25). Their ability to record multi-component biochemical information throughout various biomolecular species is an exceptional advantage. As lipids, conjugated nitrogenous bases, and cytochromes provide a strong Raman signal, whereas proteins (with their secondary structures), sugars, and nucleic acids exhibit intensive IR bands, the complementary nature of these techniques enhances their detection capabilities. Furthermore, Raman microscopy allows for subcellular resolution, depth profiling, and probing live cells, whereas an IR microscope combined with quantum cascade lasers or a focal plane detector is more easily suited for fast probing of micro-sized materials (26, 27, 28, 29, 30). The imaging modality generates images of the spatial distribution of molecular species in complex biological specimens. With the use of advanced data analysis methods segmenting and reducing hyperspectral data sets, it is possible to determine the spectral metrics of cell phenotypes, their transformations under stress and pathological conditions as well as cell–cell interactions that are used to build the models and machine learning approaches (31, 32, 33, 34, 35).

The potential of both imaging modalities for the characterization of stem cell differentiation has been reported in a few studies. An IR microscopic quality control of the differentiation of preadipocytes into adipocytes (mice 3T3-L1 cells) has indicated the recognition of early stages of differentiation (1–5 days) based on one frequency at 1739 cm^−1^ assigned to triacylglycerols. (36) A combination of single point mapping with synchrotron - IR microscopy has confirmed lipidic changes in the process of human MSC adipocyte formation at the early stages of differentiating (1–3 days) and showed the discrimination of the stages by using Principal Component Analysis (PCA) (37). Raman Surface Enhanced Spectroscopy has detected the alternation of cell morphology in a model of human AD-MSCs differentiation (38).

Herein, combining IR and Raman hyperspectral databases and data mining, we present an IRRSmetrics4stem method for dynamic monitoring of the differentiation of stem cells and its molecular heterogeneity. As a model, we chose the adipogenesis process from naïve adipose-derived mesenchymal stem cells. The method optimizes the extraction of biologically relevant information from IR and RS of the cells in the consecutive stages of differentiation by the determination of the spectral metrics and includes a machine learning (ML) approach for their prediction. While reference staining techniques are used for the recognition of lipid bodies – the main biomarker of adipogenesis, IRRSmetrics4stem can be valuable for understanding cellular changes, cellular allocation, and interactions appearing during the AD-MSCs differentiation as well as its safety controlling and efficacy of AD-MSCs in tissue engineering.

## Materials and methods

### Culturing and differentiation of adipose-derived mesenchymal stem cells

The primary cell line was used for this study, i.e., mesenchymal stem cells obtained from fat tissue (AD-MSCs) of C57BL6 mice, derived as described elsewhere (39). Only cell lines showing ≥90% expression of CD29 and CD90 markers, high CD105 markers expression, and no CD34 and CD45 expression were used for further differentiation. Cells were grown in DMEM F12 growth medium (Gibco) with the addition of 10% fetal bovine serum (FBS, Gibco) and a mixture of antibiotics penicillin/streptomycin (10,000 μg/ml penicillin, 10,000 μg/ml streptomycin; Gibco). For further experiments, cell lines from 4-6 passages were used. At a confluence of 90% the cells were washed with phosphate buffer (PBS) and then trypsinized (trypsin 0.25%, Gibco) for 3 min (37°C, 5% CO_2_). Afterward, trypsin was blocked by the growth medium. Next, the cells were centrifuged (5 min, 300 *g*) and suspended in the growth medium. Such prepared cells in the logarithmic growth phase were seeded on 24-well plates with 12 mm CaF_2_ glass slides (Crystran) at a concentration of 4 × 10^4^ cells/ml/well. Osteogenic/Adipogenic Base Media (CCM007) by StemXVivo was used as a basal medium for the differentiation process.

After 3–4 days, when the monolayer of cells was formed, the process of differentiating AD-MSCs towards adipocytes was induced. For this purpose, the differentiating medium Adipogenic Base Media + Adipogenic Supplement (CCM011, StemXVivo) was added. The medium was changed every 3–4 days. The differentiation process was stopped at selected time points (6 h, 2, 7, and 14 days; further labeled as 6 h, 2 days, 7 days, and 14 days groups) by fixing cells monolayers in 2.5% glutaraldehyde (GA at PBS, 2 h, 4°C). Samples destined for histological staining were fixed in 4% paraformaldehyde (PFA at PBS, 20 min, 24°C). Finally, after rinsing the cells twice with PBS solution, they were left at a temperature of 4°C for further research. For each time point, two biological replicates were prepared and one negative control (cells cultured in medium without the Adipogenic Supplement).

### Oil Red O staining

Oil Red O (ORO) staining was used as a reference technique. A 0.5% solution of ORO in isopropanol was prepared (Sigma Aldrich, O1391). The cells were first pre-incubated in a 60% solution of isopropanol (15 min, 24°C). Next 0.5% ORO solution was added, and the incubation was continued for 1 h (24°C). After that, the cells were rinsed four times with sterile deionized water. Microscopic observations of the cells were conducted using a Zeiss Axio Observer microscope combined with the ZEISS Axio Cam 506 color camera (100× and 200× magnification). For registered images, the ORO-stained area was counted with Gimp 2.10.14 software.

### Immunofluorescence assay

For immunofluorescence assay (IF), AD-MSCs were differentiated into adipocytes (as described above) on 4-well Lab Tek II (Thermo Fisher Scientific) coated with 0.1 mg/ml Poly-L-Lysine (Sigma Aldrich) in PBS. After each time point incubation (6 h, 2, 7, and 14 days) cells were fixed in 1.5% glutaraldehyde (GA at PBS, 1 h, 4°C), washed 0.1% BSA solution in PBS and then permeabilized and blocked with Blocker BSA in TBS (Thermo Fisher Scientific) for 1 h in RT. Anti-mFABP4 primary antibody (R&D Systems), diluted 1:50 in 0.1% BSA (Thermo Fisher Scientific) in PBS was used for staining lipids specific to differentiated cells (overnight incubation at 2–8°C). After incubation, AD-MSCs were washed with PBS, and a secondary, donkey anti-goat IgG antibody, AlexaFluor 555 conjugated (Invitrogen) was added (1 h, RT, in the dark). Again, AD-MSCs were washed with PBS and the actin cytoskeleton was stained using Phalloidin-Atto 488 (Sigma-Aldrich), diluted 1:50 in 0.1% BSA (1 h, RT, in the dark). To stain cell nuclei, BisBenzamide H33342 trichydrochloride (Sigma Aldrich, diluted 1:1000 in PBS) was used (15 min, RT, in the dark). To quench the nonspecific fluorescence, a Vector True View autofluorescence Kit (Vector Laboratories) was added (1 h, RT, in the dark). To close the Chamber Slides, Vecta Shield Virbrance—Antifade Mounting Medium with DAPI (Vector Laboratories) was used. Immunofluorescence images were acquired using a Zeiss Axio Observer microscope equipped with an Axiocam 530 monochromatic camera and ZEN Blue Edition v 3.4 (Zeiss). The size and the morphology of cell nuclei at different stages of adipogenesis were estimated by counting their number at randomly selected microscope images.

### Raman and FTIR microscopy

For Raman measurements, a WITec confocal Raman imaging system (WITec Alpha 300R Raman microscope, WITec) was employed and an excitation laser at 532 nm was used to acquire Raman spectra. The microscope was equipped with a CCD detector cooled to −80°C. Cells adhered to CaF_2_ windows and immersed in PBS solution were illuminated through a 40× water immersive Zeiss objective (NA: 1.0). To fasten Raman data collection and avoid autofluorescence, linear measurements were performed with a step size of 1 μm (10 spectra/line) and spectra were collected with an integration time of 1 s. Around 400 single spectra were acquired for each time point of the differentiation and the corresponding negative controls.

FTIR imaging was performed in transmission mode using an FTIR Agilent 670-IR spectrometer coupled with an Agilent 620-IR microscope equipped with a focal plane array detector (FPA) cooled with liquid nitrogen. FPA is a grid of detectors with dimensions of 128 × 128, thus giving 16,384 spectra at a single measurement. IR images were captured using a 15× Cassegrain objective (NA: 0.62). For such an optical setup, the field of view of a single pixel and whole detector is 5.5 μm × 5.5 μm and 700 μm × 700 μm, respectively. Single IR images were collected from an area of 1400 μm × 1400 μm providing over 65.5 thousand IR spectra. In total, the area of 164 mm^2^ was studied with 5.6 million acquired spectra for each of the differentiation phases and corresponding negative controls. FTIR spectra were gathered in the spectral range of 4000-900 cm^−1^ with a spectral resolution of 4 cm^−1^ by co-adding 128 and 256 scans for sample and background, respectively.

### Spectral preprocessing

Raman spectra were initially preprocessed by using a cosmic ray removal filter with a size of 3 and a dynamic factor of 8 (Project Five software, WITec 5.0). For baseline correction, the third-grade polynomial was used. Next, spectra were extracted as single files, and further data preprocessing was continued in OPUS 7.0 software. Raman spectra were then truncated in the spectral region of 700–3050 cm^−1^, baseline-corrected (10 iterations), smoothed according to a Savitzky-Golay protocol (9 points), and vector normalized. Next, outliers were rejected, and the spectra were finally averaged.

FTIR data preprocessing started using CytoSpec (ver. 2.00.04) (40) and MatLab (R2017a, Natick) software. Working directly on the imported IR images, the process included reduction of noise according to a PCA algorithm (13 PCs), cutting the spectral region to 920-3700 cm^−1^, and smoothing (Savitzky–Golay algorithm, 13 pts). In the next step, regions of interest (ROIs) in the images were selected based on the high signal-to-noise ratio (S/N) estimated in the spectral region of 1620–1680 cm^−1^ (the protein distribution). From a single IR image (an area of 1400 μm × 1400 μm), four ROIs were determined. The spectra included in the single ROI (area of 700 μm × 700 μm) were averaged and then exported. As a result, 40 mean spectra were obtained per the experimental group. Further data preprocessing was continued in OPUS 7.0 software. Initially, a straight line in the range of 2400-2200 cm^−1^ was generated to remove the effect of the CO_2_ band. In the next step, second derivative spectra were calculated (13 points), vector-normalized, and averaged.

### Semi-quantitative and multivariate analysis

Integral intensities of Raman and IR bands were calculated as an area under the band contour (OPUS 7.0) of single and ROIs extracted spectra, respectively. Then, their box charts were constructed. An analysis of variance was performed using the ANOVA statistical model, while significance (*P*-values) was determined by a Tukey’s test on three levels of statistical significance (*P* < 0.05; *P* < 0.01; *P* <0.001) (Origin 2021b).

Hierarchical cluster analysis (HCA) was performed on the processed second derivative FTIR spectra in the bio-region (3050-2800 cm^−1^ and 1750-920 cm^−1^) (OPUS 7.0). Distances between objects were calculated according to a Euclidean method while grouping was computed by using a Ward algorithm were used. Results were presented as dendrograms.

Principal Component Analysis (PCA) was performed by using Unscrambler X 10.3 software (CAMO Software AS., Norway). Before analysis, Raman spectra were smoothed (Savitzky–Golay, third-order polynomial, 9 pts) while FTIR spectra were transformed into a second derivative (Savitzky–Golay, second-order polynomial, 13 pts). Next, all spectra were baseline corrected (offset) and vector normalized. PCA was performed in the bio-regions of 950–3050 and 700-3050 cm^−1^, for mean-centered FTIR and Raman spectra, respectively, with a Singular Value Decomposition (SVD) algorithm of cross-validation and 7 principal components. Scores and loadings graphs were generated to show grouping and variance within the Raman and FTIR spectral databases.

Partial Least Square regression (PLSR) was performed on FTIR data sets pre-processed like for PCA (Unscrambler X 10.3 software). Ca. 80% spectra from the first biological replicate were used to calibrate and validate models using a NIPALS algorithm with full cross-validation and 10 factors. Then, the models were tested on the remaining 20% of the dataset. The same models were used in the classification of the adipogenesis phases prepared in the second biological replicate.

**Scheme 1:**
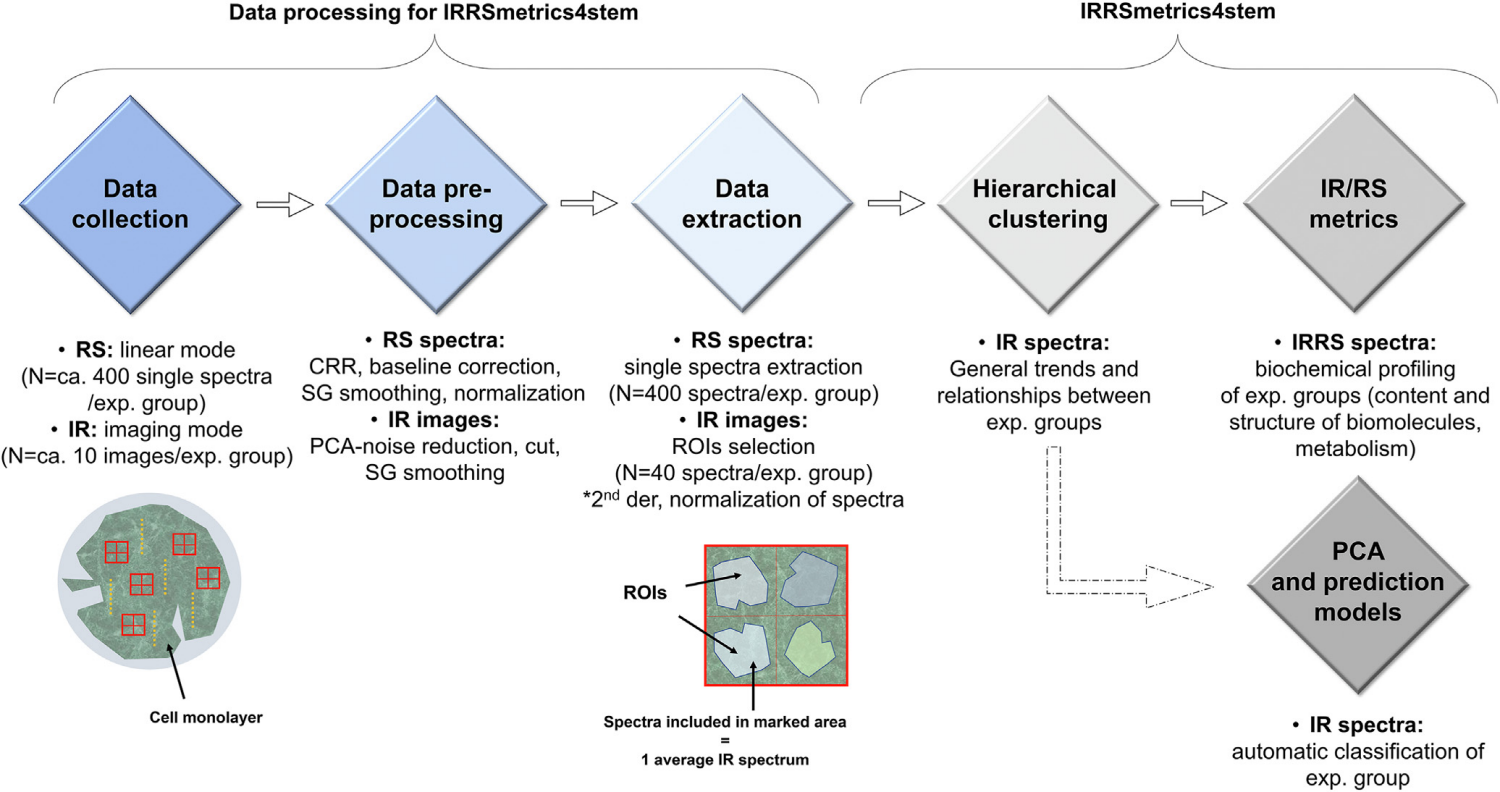
The flowchart representing data processing for IRRSmetrics4stem approach.

All graphs showing spectra, semi-quantitative analysis, and chemometric analysis were prepared using Origin 2021b software. The flow chart summarizing data processing for IRRSmetrics4stem approach is illustrated in Scheme 1.

## Results

**Fig. 1:**
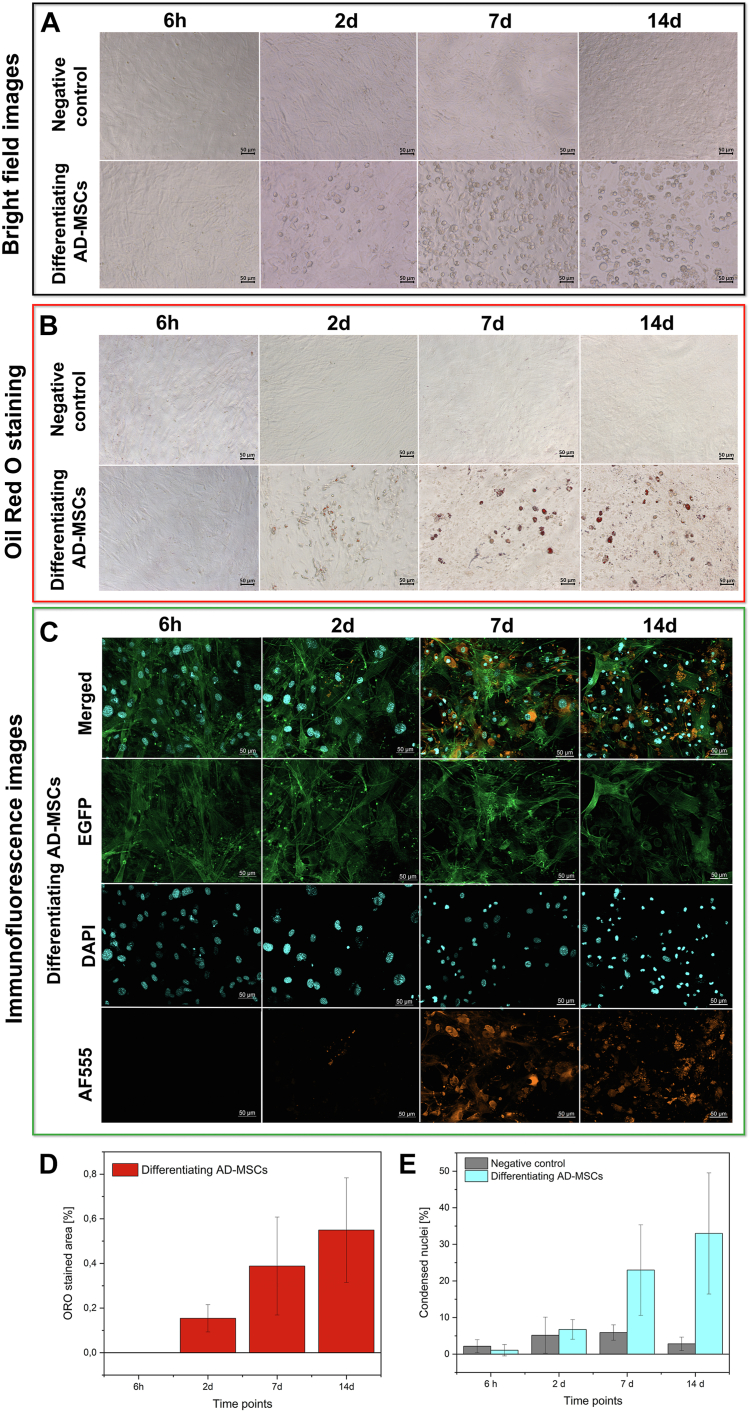
AD-MSCs undergoing differentiation and the control cell culture (Negative Control; NC). A: bright field images. B: oil Red staining (ORO) showing lipid droplets stained in red (magnification 200×). C: immunofluorescence images: EGFP (green) – actin cytoskeleton; DAPI (blue) – nuclei; AF555 (orange) – lipids (magnification 200×); Images for NCs are displayed in supplemental Fig. S1. D: the percentage of area stained with ORO for subsequent phases of adipogenic differentiation. E: the percentage of condensed-like, small-size nuclei for subsequent stages of the adipogenic differentiation in comparison to control cells.

To confirm the proper course of induced AD-MSCs adipogenesis, the amount of lipid droplets (LDs) is commonly elucidated by Oil Red O and immunofluorescence staining (Fig. 1A–C). Their content directly refers to the phase of differentiation. Another feature of progressing adipogenesis is a characteristic modification of the actin cytoskeleton and the size of nuclei associated with modifications within the nuclear structure. This evolution during differentiation is illustrated in Fig. 1C, E.

The bright field images show an alteration in the morphology of the AD-MSCs monolayer at a relatively early phase, as some aggregations appeared on day 2 and steadily increased over time (Fig. 1A). The same aggregations are stained in red with the ORO method demonstrating that these are lipid droplets (Fig. 1B). At day 7, LDs become more visible, and their number increases significantly in most cells triggered for adipogenesis, like on the 14th day of the experiment. Markedly enlarged and merged LDs in differentiating cells give the unambiguous identification of adipocytes on the morphological ground. The percentage of stained area also increases gradually for the consecutive phases of the adipocyte formation (Fig. 1D). In all negative control groups and after 6 h of differentiation, there are no signs of the adipogenesis process throughout the whole period of observations. Importantly, the ORO approach is only applicable to detect neutral lipids such as triacylglycerols and cholesterol esters (CEs), so the observable stained area is directly dependent on the chemical composition of lipid droplets (41). Therefore, the estimated and actual area of LDs in the sample could vary. More precise outcomes can be achieved with the immunofluorescence approach. The AF555 staining reveals the LD formation of various sizes that occupy the major area of the cell monolayer from the seventh day of the differentiation (Fig. 1C). The main feature of adipogenesis, i.e., lipid droplet formation, is coordinated with modifications of the actin cytoskeleton and the size and the morphology of cell nuclei appearing as condensed-like and small-size structures in contrast to the nuclei in non-differentiated AD-MSCs (Fig. 1C, E). The IF images of control AD-MSCs are displayed in Supplemental Information (see supplemental Fig. S1).

**Fig. 2:**
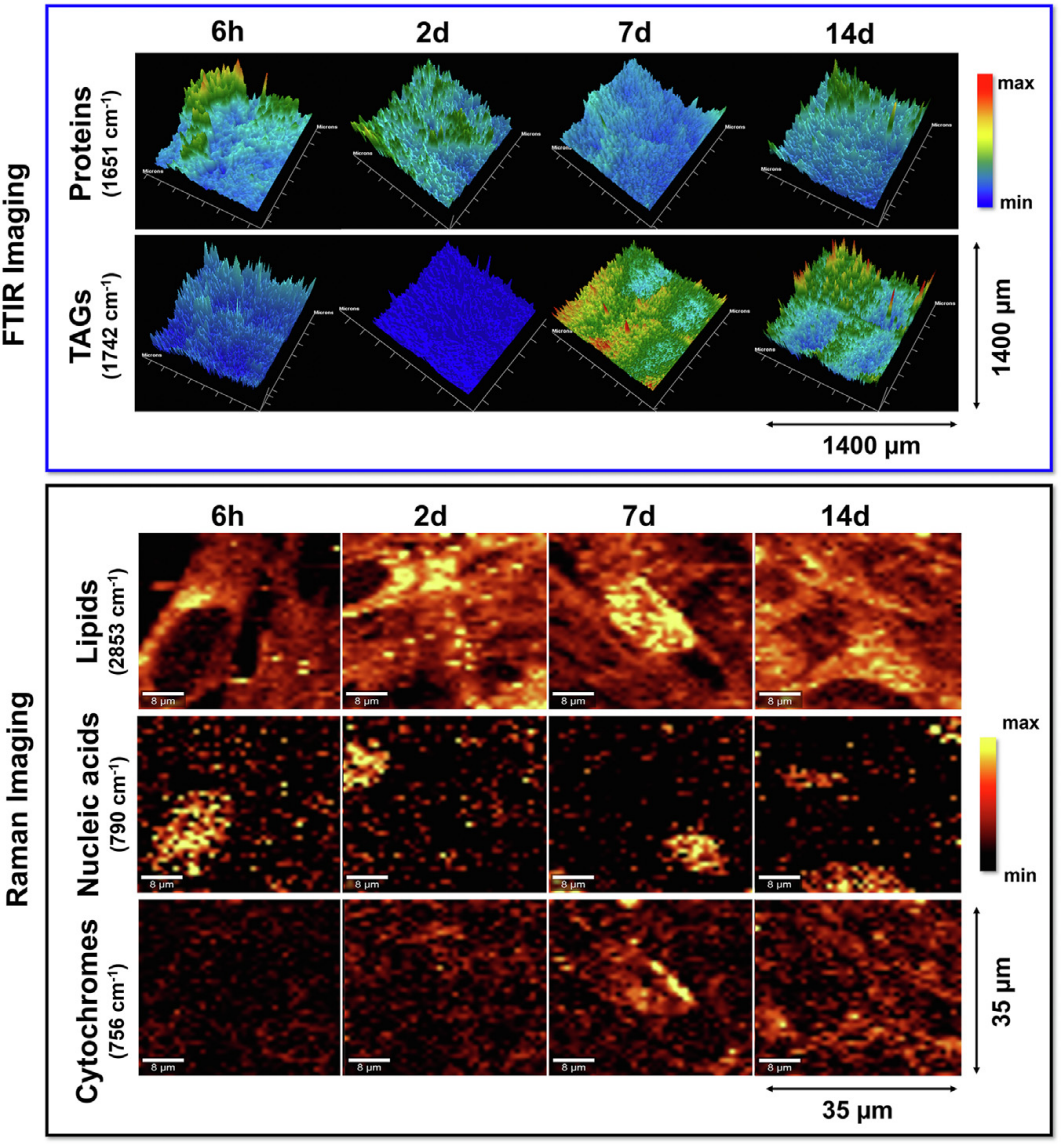
Exemplary chemical maps showing the distribution of proteins (IR: 1651 cm^−1^), triacylglycerols (IR: 1742 cm^−1^), lipids (RS: 2853 cm^−1^), nucleic acids (RS: 790 cm^−1^), and cytochromes (RS: 756 cm^−1^) in the consecutive phases of AD-MSCs adipogenesis. The maps were constructed based on integral intensities of the marker IR and RS bands.

A similar visualization of biochemical composition across the sample of the cell monolayer can be illustrated by the distribution maps constructed from IR and RS images (Fig. 2) We show their examples for protein and lipid components to highlight the opportunity for the label-free monitoring of their presence and co-localization by spectroscopic imaging. In this work, we omit the examination of these maps because this approach is effective in the case of a priori known spectral marker, e.g., the 1742 cm^−1^ band of TAGs.

### Determination of spectral metrics for the adipogenesis process

**Fig. 3:**
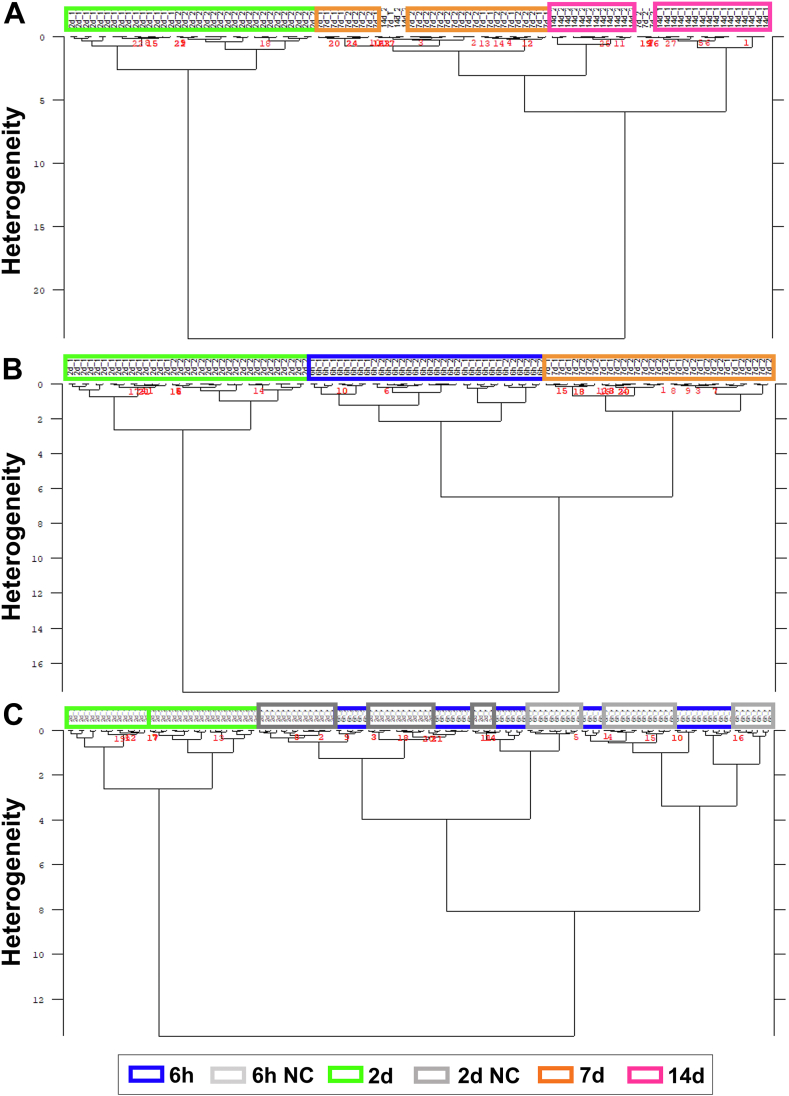
HCA dendrograms calculated for the second derivative FTIR spectra in the regions of 3050-2800 cm^−1^ and 1750-920 cm^−1^. Clustering of the adipogenesis phases at selected time points after its induction: (A) 2, 7, and 14 days; (B) 6 h, 2 and 7 days; (C) 6 h and 2 days with the corresponding negative controls (NC).

Through the FTIR imaging of cell monolayers, a vast examination of a large AD-MSCs population is possible. The obtained hyperspectral database is used to reveal alternations in the biochemical composition of samples during ongoing adipogenesis. As a first step of data analysis, the hierarchical cluster analysis (HCA) is performed to examine the occurrence of general trends and relationships between the consecutive stages of the process of differentiating AD-MSCs towards adipocytes. The obtained dendrograms are displayed in Fig. 3.

**Fig. 4:**
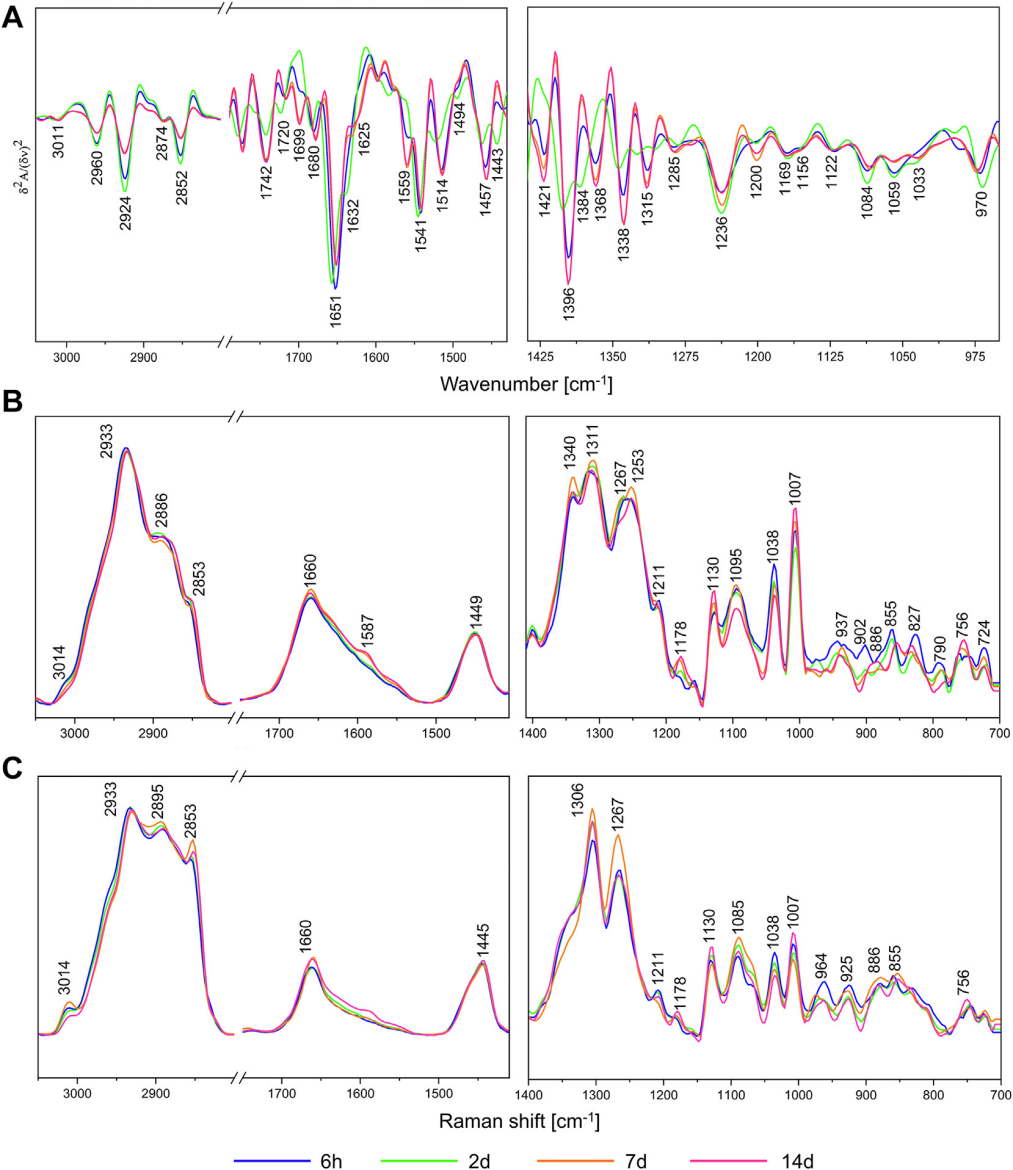
Averaged vibrational spectra from subsequent stages of adipogenic differentiation. A: second derivate FTIR spectra. B: Raman spectra – proteinaceous profile. C: Raman normal spectra – lipidic profile. Plots represent two spectral regions of 3050-1410 cm^−1^ (*left*) and 1400-700 cm^−1^ for RS and 1400-950 cm^−1^ for FTIR (*right*).

These dendrograms show the overall biochemical heterogeneity between the adipocyte maturation phases and indicate their separation. The 2-day group establishes a separate branch of the dendrogram with a big heterogeneity rate over 20, indicating its very distinct spectral profile compared to the 6 h and later phases (7 and 14 days) of adipogenesis (Fig. 3A). Since several spectra of the 7-day and 14-day groups are grouped, their biochemical profiles are similar. The HCA analysis of the 6 h, 2 day, and 7 day groups confirms the unique spectral features of the AD-MSC cells exposed to the differentiation medium for 2 days (Fig. 3B). Furthermore, cluster analysis of two early phases of adipogenesis and their corresponding negative controls shows that the 6 h and NC groups are assigned to one class and mixed within sub-classes, whereas the 2 days cells are separated from them (Fig. 3C). Therefore, one can draw a few conclusions from this simple data mining method of the IR spectra, i.e. 1/the significant biochemical transition is not started yet after 6 h, 2/the predominant biochemical changes observed in the cell monolayers after 48 h of cultivation result from the activation of adipogenesis, and 3/cell proliferation has a little impact on AD-MSCs' transformation since NCs exhibit different spectral properties. The latter is confirmed by the HCA analysis of each differentiation phase with its negative control (supplemental Fig. S2). This part of IRRSmetrics4stem is relatively short and does not require sample preparation. After ca. 5 h of data collection per experimental group and automatic basic pre-processing (using a standard IR microscope), the reduced data sets are prepared by the selection of high S/N ratio spectra and their averaging for randomly selected ROIs (here, in total 18 mm^2^) to cover up to 20% of the cell monolayer. The dendrograms are immediately calculated based on 1080 spectral variables by using standard clustering methods. The first observable changes in the cell morphology are found already on day 2 of differentiation in ORO and IF staining (Fig. 1B–D) and the HCA dendrogram also separated these phases of adipogenesis. Since only single LDs appeared in ORO, FTIR spectra from day 2 must indicate other biomolecular transformations accompanying triggering adipogenesis. To investigate it, we propose the second step of the IRRSmetrics4stem method. First, the averaged FTIR and RS spectra of each differentiation phase are examined, and their bands show the presence of key biomolecules such as proteins, lipids, and nucleic acids (Fig. 4 and supplemental Tables S1–S3). To fasten Raman data collection, we acquired ca. 400 single Raman spectra per the experimental group instead of a time-consuming high-spatial Raman imaging of single cells shown in Fig. 2. The latter is particularly difficult for cell monolayers because of tight junctions between cells and autofluorescence. To assess the heterogeneity among the Raman spectra, Principal Components Analysis (PCA) indicates their variety and here we identify proteinaceous and lipidic Raman profiles (Fig. 4B, C and supplemental Table S3). The first one is attributed to the cytoplasm, nucleus, and perinuclear area whereas the second one is typical for the accumulated lipid bodies (33, 34, 42). Despite the random selection of spots for the Raman measurements, the number of the lipidic signatures of the differentiated AD-MSC cells is greater than for NCs and it increases in the consecutive phases of adipogenesis (supplemental Table S3).

The interpretation of IR and Raman bands gives an insight into the biochemical composition of the experimental groups and suggests first spectral metrics that can be selected for further semi-quantitative analysis. A visual inspection of the FTIR spectra suggests that the content of all cellular lipids increases up to two days of differentiation (CH_2_ vibrations at 2924 and 2852 cm^−1^), c.f. Figure 4A. The 2960 cm^−1^ band (CH_3_ vibrations) may indicate the alternation of the length of the lipid acyl chains. Interestingly, intensities of 1742 and 1514 cm^−1^ bands (assigned to TAGs and tyrosine residue, respectively) are significantly reduced on day 2 whereas the content of fatty acids (1720 cm^−1^) and β-sheet secondary structures of proteins (1632 cm^−1^) increases. A unique marker of the 2 days group is found at 1421 cm^−1^ (Fig. 4A). This IR signal attributed to α conformation of the acyl groups (α-CH_2_) in fatty acids disappears whereas IR markers of nucleic acids (1236, 1084, and 970 cm^−1^) are significantly elevated. In turn, the averaged Raman spectra of the dominant proteinaceous nature reveal major molecular changes in the AD-MSC differentiation from the seventh day (Fig. 4B). Increased intensities of the 1585, 1130, and 750 cm^−1^ bands of cytochromes indicate enhanced cell metabolism. Interestingly, the ratio of cross-linked (1038 cm^−1^) and total phenylalanine (1007 cm^−1^) drastically changes during adipogenesis reaching a high level after 14 days of differentiation. Components such as fatty acids (FA, 1340 cm^−1^) and nucleic acids (G, 1311 cm^−1^) are synthesized. The Raman spectra with the lipidic profile show a high content of long-chain fatty acids and TAGs in the lipid aggregates at day 7 (2853 and 1306 cm^−1^, respectively) and their unsaturation increases rapidly (3014 and 1267 cm^−1^) (Fig. 4C).

### Metrics for lipids

**Fig. 5:**
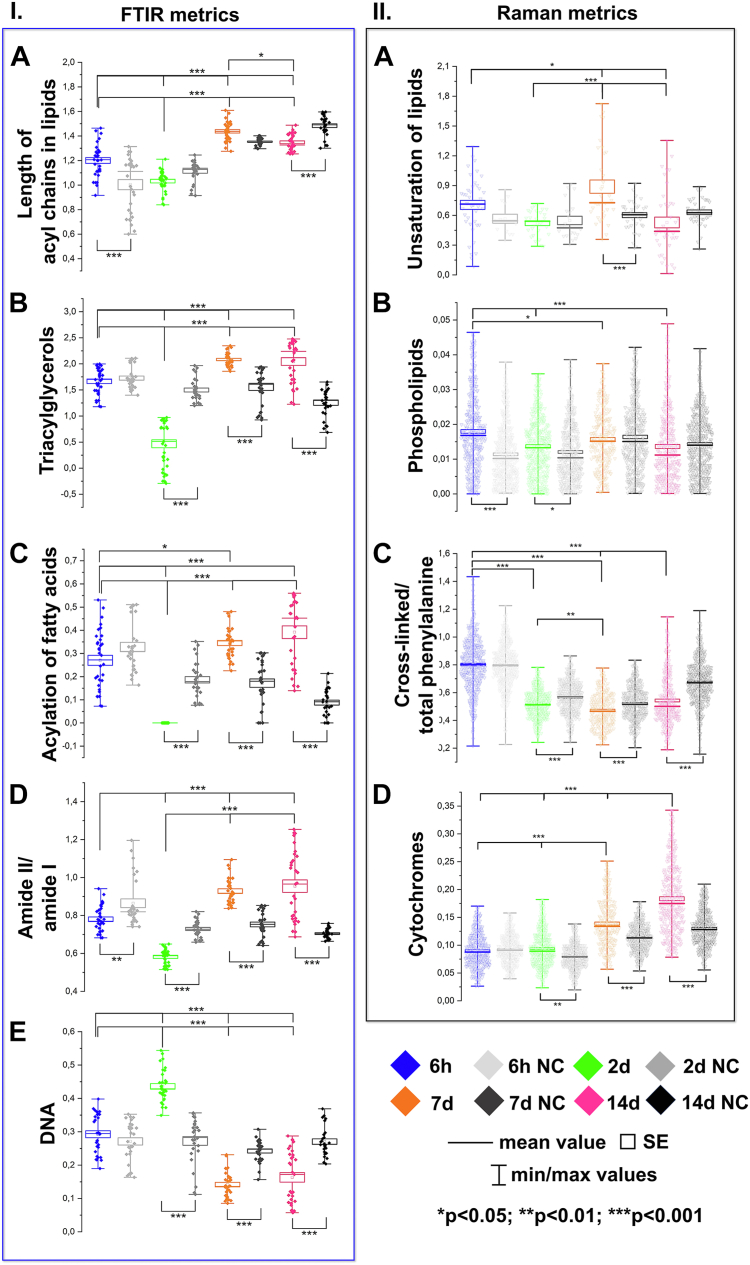
Box diagrams representing semi-quantitative analysis of biomolecules determined from the selected bands. Panel I. FTIR metrics: (A) shortening of acyl chains in fatty acids [(2989-2944 cm^−1^)/(2868-2837 cm^−1^)]; (B) triacylglycerols (1761-1727 cm^−1^); (C) acylation of fatty acids chains (1430-1410 cm^−1^); (D) alternation of secondary structures in proteins [(amide II/amide I; 1595-1482 cm^−1^)/(1708-1609 cm^−1^)], and (E) DNA (990-949 cm^−1^). Panel II. Raman metrics: (A) unsaturation of lipids [(1280-1250 cm^−1^)/(1320-1285 cm^−1^); (B) phospholipids (730-715 cm^−1^); (C) ratio of cross-linked and total phenylalanine amino acid residue [(1050-1026 cm^−1^)/(1020-994 cm^−1^)], and (D) cytochromes [(1595-1575 cm^−1^)+(1140-1120 cm^−1^)+(760-740 cm^−1^)]. (A) calculated from the RS spectra with the lipidic profile, (B), (C), and (D) calculated from the RS spectra with the proteinaceous profile.

The integral intensities of the IR and RS bands and their ratios deliver a set of spectral metrics indicating molecular changes during the AD-MSC adipogenesis (Fig. 5). The ratio of CH_3_ to CH_2_ vibrations (IR: 2960/2852 cm^−1^) indicates the shortening of the length of the acyl chain in fatty acids (Fig. 5IA). Here, the biosynthesis of long-chain FAs is maintained to day 2, and then it is switched to the production of shorter FAs. In the case of the corresponding NCs, a gradual shortening of the FA acyl chains is observed over time indicating that we can detect subtle changes most probably related to the prolonged cell culture itself. In addition, the lipids accumulated in LDs are the most unsaturated on day 7 as revealed by the Raman ratio of the =CH and CH_2_ moieties (RS: 1267/1306 cm^−1^) (Fig. 5IIA). The unsaturation degree of the differentiating and negative control cells does not differ for up to two days and drops on day 14, so it is an exclusive marker for the first signs of the LD aggregation observed by ORO and AF555 staining (Fig. 1B, D).

The content of triacylglycerols (IR: 1742 cm^−1^) significantly decreases on day 2 and next they are synthesized in the aggregated LDs (Fig. 5IB). There is no doubt that this metric is a key element for monitoring adipogenesis because its alternation is not observed in NCs over the time of the experiment. The 1421 cm^−1^ band (IR) assigned to conformational changes in the acyl chains of fatty acids shows a similar trend to TAGs (Fig. 5IB, C). The absence of this band is correlated with the formation of the lipid bilayer in the cellular membrane as reported in a model IR study (43). This spectral marker reveals a dramatic change in the conformation of the acyl chains within sn-1 and sn-2 positions around the glycerol skeleton in TAGs. Thus, its disappearance on day 2 in the differentiating AD-MSCs can be a key marker of these conformational modifications and the starting point of the formation of lipids droplets as we observed in ORO and IF staining (Fig. 1B, C). In the late phases of adipogenesis (7^th^ and 14^th^ days), the level of this spectral marker is higher than for the 6-h cell culture suggesting a different rearrangement of lipids in the well-formed LD membranes. In the case of NCs, a gradual decrease of this band absorbance might be correlated with cell aging and degradation of intracellular membranes during prolonged cell culture. A Raman metric for phospholipids (724 cm^−1^) - a main component of the cellular membranes, decreases slightly during both differentiation and prolonged culturing of AD-MSCs (Fig. 5IIB).

### Metrics for proteins

Modifications of secondary structures of proteins are monitored by the ratio of the amide II and amide I bands in the IR spectrum (Fig. 5ID) (44, 45, 46, 47). The most significant structural reorganization of this class of biomolecules is observed on day 2 of adipogenesis, and it is accompanied by the formation of β-sheets – a first sign of protein aggregation (the 1632 cm^−1^ band, Fig. 4A). In our previous IR studies on the oxidative stress of erythrocytes, the alternation in this ratio was attributed to the aggregation of the protein membranes when accompanied by the appearance of the 1625 cm^−1^ band as it is observed here for days 7 and 14, Fig. 4A (44, 48). No significant alternations of protein conformations are found in NC throughout the time of the experiment (Fig. 5ID). Two Raman signals assigned to vibrations of the Phe residue (1038 and 1007 cm^−1^) indicate their participation in cross-linking of proteins, particularly important for the stability of protein hydrophobic interior in the cellular cytoskeleton (49, 50). Here, we observe a statistically significant gradual decrease in the ratio of these bands for the stages with LDs visible in staining (Figs. 1B, C and 5IIC). Since the value of this marker for the differentiated mesenchymal stem cells is lower than for NC, the intracellular membranes and/or cytoskeleton in AD-MSCs undergo important transformations to constitute adipocytes (see Fig. 1C and supplemental Fig. S1).

Certainly, the metabolism of the cells must be affected by the differentiation of AD-MSCs into adipocytes. We observe this phenomenon based on the Raman signature of mitochondrial cytochromes (1587, 1130, and 756 cm^−1^). We note that firstly the differentiating AD-MSC and NC cells amplify their metabolism, supposedly due to their proliferation, between days 2 and 7 (Fig. 5IID). Then, the biosynthesis of lipids and their accumulation in LDs additionally up-regulates cellular activity compared to NC.

### Metrics for DNA

The DNA metric is proposed by using an IR band at 970 cm^−1^ whose high intensity indicates that chromatin is decondensed. This DNA parameter considerably changes over the differentiation process, reaching the highest value on day 2 (Fig. 5IE). We propose here that DNA replication is boosted on day 2 and significantly suppresses when pre-adipocytes are formed from the mesenchymal cells (7 and 14 days) even below the level determined for the negative control. In the latter, the DNA synthesis is maintained at a similar level within 14 days. This observation is congruent with DAPI staining of the cells (Fig. 1C, E and supplemental Fig. S1). The first observation of remodeling of nuclear architecture is noted on day 2: the size of single nuclei varies compared to the 6 h group. Afterward, the shrinking of the nuclei is noticeable.

### Discrimination and prediction models

**Fig. 6:**
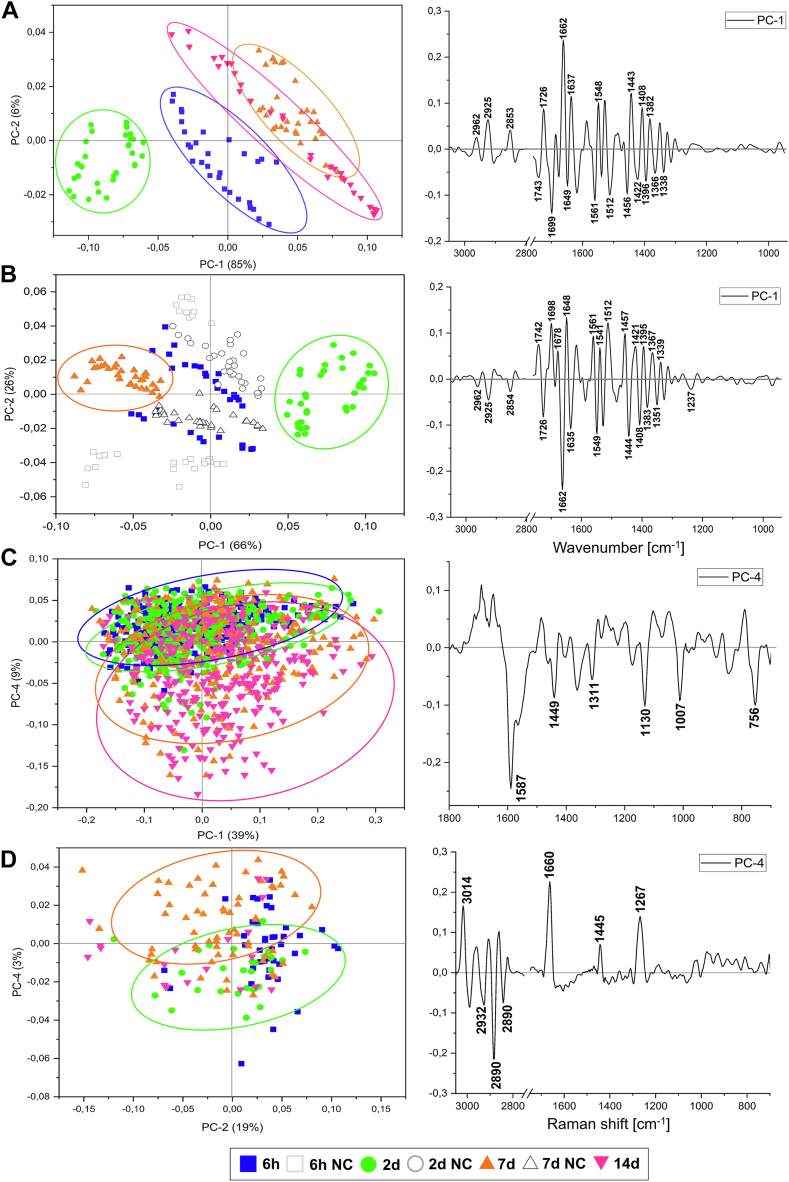
The scores (*left*) and loadings (*right*) plots from Principal Component Analysis performed on (A), (B) FTIR spectra (3050-950 cm^−1^); (C) proteinaceous Raman spectra (1800-700 cm^−1^), and (D) lipidic RS spectra (3050-700 cm^−1^) collected from the investigated experimental groups of the differentiated AD-MSCs. Each point corresponds to a single spectrum.

Principal Component Analysis (PCA) is a perfect unsupervised chemometric tool for the evaluation of data sets in terms of their usefulness for a machine learning approach that is the final step of IRRSmetrics4stem. The score plots represent the separation of cells from consecutive stages of adipogenic differentiation, while the loading plots exhibit the spectral discriminators leading to the mentioned segregation. Here, for the visualization of loading plots, we chose only those principal components that directly indicated the grouping of cell classes. PCA analysis of FTIR data for AD-MSCs cultured in the differentiating medium shows clear-cut segregation of 6 h, early (day 2), and late (days 7 and 14) groups along PC-1 and 2 with a total variation of 91% (Fig. 6A). This result agrees well with the semi-quantitative analysis in Fig. 5. The main discriminators of PC-1 appear at 1662, 1560, 1549, 1443, and 1396 cm^−1^ (assigned to proteins) and at 2853, 1742, 1726, and 1423 cm^−1^ (assigned to lipids). We repeated PCA analysis for the key time points including their negative control (Fig. 6B). PC-1(−) groups 6 h and NC samples separating them from the early (2days) and late (7days) stages of adipogenesis with a variance of 66% like in the HCA dendrogram (Fig. 3C). The loadings plot shows a similar set of spectral discriminators. In turn, principal component analysis of the Raman spectra with the proteinaceous features of the differentiated cells does not show such a strong separation of the experimental groups as FTIR spectra (Fig. 6C). The scores plot for PC-1 and 4 indicate a distinct biochemical composition of the cells from the late stages. The most significant loading vectors are assigned to cytochromes (1587, 1311, 1130, and 756 cm^−1^) and Phe (1007 and 1449 cm^−1^) confirming our findings in the semi-quantitative analysis (Fig. 5IIC, D). In turn, the scores plot calculated for the RS spectra of the lipidic specifics confirms a high unsaturation degree of lipids in LDs on day 7 only (3014, 1660, 1267 cm^−1^) (Figs. 5IIA and 6D). As mentioned above, Raman spectra were acquired from randomly selected spots of the cellular films preventing their assignment to the cellular compartments.

PCA results indicate that FTIR data sets are suitable for the classification of the adipogenesis phases. For this purpose, we employ Partial Least Square Regression (PLRS) with a full cross-validation method. We prepared three models to distinguish 1/the 6 h group from the early phase (day 2) to identify the process that triggers the formation of adipocytes, 2/early versus late phases (day 14) to assess if the late differentiated AD-MSCs completed their differentiation, and 3/the early phase from its negative control. The obtained parameters of the regression models for calibration are very good with an R-square above 0.94 (Table 1). Then, the models are validated on the independent in vitro experiment. The true positive rate is 100% for models 1/and 3/ while 91% of IR spectra are correctly classified as the early phase in model 2 (Table 1). Graphs for the calibration and classification models of the independent in vitro experiment replicate are displayed in supplemental Figs. S3 and S4.

**Table 1:** Results of PLSR models for their calibration, validation, and prediction

Model	Samples (N)		RMSEC/R^2^C	RMSEV/R^2^V	True Positive Rate
	Model	Classification			
1/6 h versus 2 days	6 h = 30 2 days = 33	6 h = 28 2 days = 16	0.247/0.939	0.288/0.920	91%
2/2 days versus 14 days	2 days = 31 14 days = 31	2 days = 16 14 days = 42	0.085/0.993	0.097/0.991	100%
3/2 days versus 2 days NC	2 days = 35 2 days NC = 20	2 days = 16 2 days NC = 20	0.238/0.942	0.277/0.924	100%

RMSEC, Root Mean Square of Error for calibration; R^2^C, R-square of calibration; RMSEV, Root Mean Square of Error for validation; R^2^V, R-square of validation.

## Discussion

Adipogenesis is an extremely dynamic process of cell differentiation in which AD-MSCs transform into mature adipocytes (51). After initial proliferation and growth, AD-MSCs are transformed into preadipocytes. This means that concurrently, they lose their potential to differentiate into other types of mesenchymal cells, and since then, their pathways of differentiation have been determined and cannot be changed (52, 53). These cells undergo the so-called clonal expansion. They undergo numerous mitotic divisions performed to rapidly increase the final number of cells which then differentiate into adipocytes. Relatively early preadipocytes modify their shape and physiology by altering their gene expression, leading to different protein content and structure, allowing full transformation into adipocytes. These changes manifest by morphological alterations demonstrated here by the change of the cell shape, lipid droplets’ appearance, the important modification of the actin cytoskeleton (54, 55, 56, 57), and nuclear size and morphology (58, 59).

**Scheme 2:**
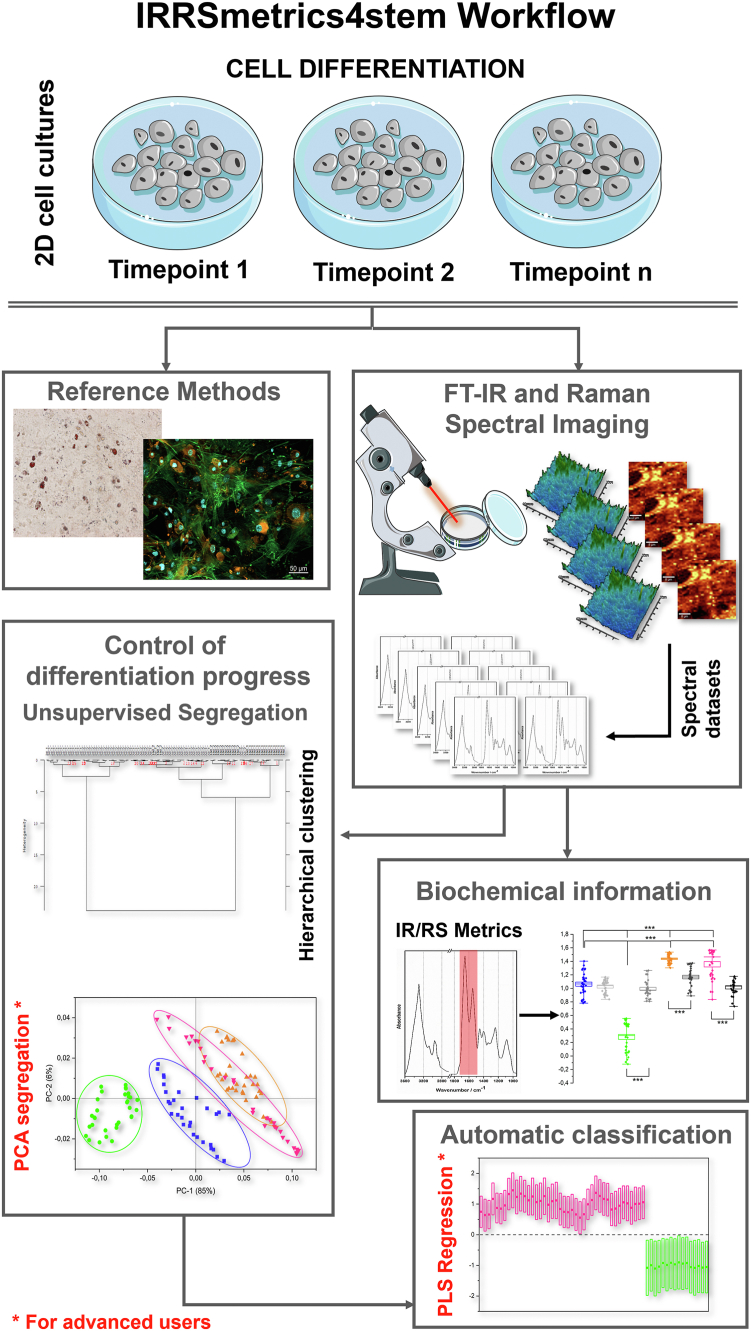
The workflow of the developed IRRSmetrics4stem approach.

IRRSmetrics4stem proposed here delivers critical findings that provide insights into the chemism of the early stages of adipogenesis. Through the study of cell monolayers, which includes the measurement and analysis steps, we developed a label-free approach for investigating cells and producing this chemical complementarity summarized in Scheme 2. We demonstrate that, even though conventional bioimaging techniques only display morphological characteristics, these methods distinguish the molecular and metabolic variations between the subsequent stages of adipocyte formation.

The analysis of the IR and RS bands allowed us to identify potentially the most relevant spectral markers of the whole adipogenesis process. Clustering and principal component analysis of FTIR spectra indicate distinct spectral profiles; here on day 2 (Figs. 3 and 6A, B). The selected spectral metrics can be used for monitoring the metabolic reprogramming of AD-MSCs during adipogenesis. The most pronounced differences between the experimental groups show the first stimuli to form pre-adipocytes and then the cell metabolism is regulated toward the production of lipid droplets. The observed increase in DNA and the occurring reorganization of cell protein structures indicate an increased proliferation of cells cultivated in the differentiating medium (Fig. 5ID, E). The reorganization of protein structures and conformational rearrangement associated with the formation of LD membranes can be observed by the signals of Phe and FA acylation (Fig. 5I-IIC). Then after the proliferation period for later stages at day 7 and 14, a significant increase in lipid content is observed, as confirmed by spectroscopic markers of lipid synthesis (1742 cm^−1^). Also, the composition of lipids varies as their unsaturation level increases at day 7 (Fig. 5IIA). Additionally, as the adipogenesis process is highly endo-energetic, the RS spectra reveal the high contribution of cytochromes (1587, 1130, and 756 cm^−1^) from day 7 (Fig. 5ID). This is equivalent to the onset of the process of preadipocyte formation and is fully consistent with biological observations since the processes of proliferation and differentiation do not occur simultaneously (52, 53). Our observations suggest three stages of AD-MSC adipogenesis, i.e. 1) a preparatory phase (initial stage) comprising the period up to 2 days of differentiation, 2) the phase of cell transformation (clonal expansion) observed on day 2, and 3) the stage of cell preadipocytes formation and maturation into fully functioning adipocytes after day 2. The spectral metrics are statistically significant at min. level of *P* < 0.05, so the randomness of the observed trends is excluded. Some phases, on day 14, show markedly increased heterogeneity within the spectra as is expected because of the high dynamics and complexity of adipogenesis (Fig. 6). Simply, the cells may vary and mature at different rates. We also show the high efficiency of the prediction (over 90%) of the differentiation progress based on PLSR analysis (Table 1). These preliminary models constitute a foundation for the further development of the automatic AD-MSCs’ stage classification and can be applied for other types of cell differentiation now.

The IRRSmetrics4stem method highly correlates with the reference ORO and IF staining (Fig. 1B, C) as the first visible changes in cell monolayers’ morphology are observed on day 2 of differentiation. On day 14, LDs are markedly enlarged and merge into larger clusters, which gives the possibility of unambiguous identification of adipocytes. This means that vibrational features and their analysis provide valuable biochemical information about the process under investigation, thereby complementing standard clinical techniques.

## Conclusion

We report the IRRSmetrics4stem protocol, based on label-free non-destructive molecular spectroscopy, to identify cell chemistry during the differentiation process of AD-MSCs differentiation to mature adipocytes as an example. It is proven here that not only histological staining but also vibrational spectroscopy, is still able to track lipid alternations in adipogenesis. To our knowledge, this method represents the first tentative to describe these changes during the specifically induced direction of cell differentiation. As such, it paves the way to characterize the chemistry of other processes of cell differentiation and delivers critical insight into the holistic view of molecular changes taking place during cell differentiation. To our best knowledge, it is the first report of this kind, which might initiate the label-free recognition of chemical processes involved in cell differentiation. Our concept was to show that specific IR and Raman bands infer known biological processes as well as reveal those that require the use of sophisticated tools and assays. We also envision another pathway for the use of IRRSmetrics4stem by non-specialists in the interpretation of vibrational spectroscopy. A high throughput monitoring of adipocyte formation daily is also possible by simple measurements, minimal sample preparation, and automatic machine learning methods as we showed in this work. IR and RS microscopy, fibrous optics combined with these spectrometers as well as handheld instruments gain popularity in clinical and biological laboratories and IRRSmetrics4stem needs to be only translated into a desired technological solution. The analytical protocol developed here can be considered an innovative approach, as it significantly approximates the actual state of the biological material studied and delivers novel information on the chemistry of cell differentiation.

## Data availability

The datasets generated during and/or analyzed during the current study are available from the corresponding author on reasonable request (Kamilla Malek, Jagiellonian University in Krakow, kamilla.malek@uj.edu.pl).

## Supplemental data

This article contains supplemental data (24, 31, 32, 43, 49, 60, 61, 62, 63, 64, 65, 66, 67, 68, 69, 70, 71).

## Conflict of interest

The authors declare that they have no known competing financial interests or personal relationships that could have appeared to influence the work reported in this paper.
