# Supplementary material for: Adipose-derived mesenchymal stem cells' adipogenesis chemistry analyzed by FTIR and Raman metrics
Source: J Lipid Res. 2024 Jun 4;65(7):100573. doi: 10.1016/j.jlr.2024.100573 (PMC11260339; doi:10.1016/j.jlr.2024.100573)
Supplement: Supplemental Information [file mmc1.docx]

*Supplemental information*

**Adipose-Derived Mesenchymal Stem Cells' adipogenesis chemistry analyzed by FTIR and Raman metrics**

Karolina Augustyniak^1,2,˘^, Monika Lesniak^3,˘^, Hubert Latka^1^, Maciej P. Golan^3,4^, Jacek Z. Kubiak^3,5,*^, Robert Zdanowski^3,*^, Kamilla Malek^1,*^

^1^ Department of Chemical Physics, Faculty of Chemistry, Jagiellonian University in Krakow, Gronostajowa 2, 30-387 Krakow, Poland

^2^ Doctoral School of Exact and Natural Sciences, Jagiellonian University in Krakow,
prof. S. Lojasiewicza 11, 30-348 Krakow, Poland

^3^ Laboratory of Molecular Oncology and Innovative Therapies, Military Institute of Medicine – National Research Institute, Szaserow 128, 04-141 Warszawa, Poland

^4^ Institute of Psychology, The Maria Grzegorzewska University, Szczesliwicka 40, 02-353 Warsaw, Poland

^5^ Dynamics and Mechanics of Epithelia Group, Institute of Genetics and Development of Rennes (IGDR), Faculty of Medicine, University of Rennes, CNRS, UMR 6290, 35043 Rennes, France

**Table S1.** FTIR band positions and their assignments to vibrational modes of biomolecules (1–9).

| *Band position [cm^-1^]* | *Assignment to biomolecules and vibrational modes* |
| --- | --- |
| 970 | DNA; ν(C-N-C) |
| 1033 | Carbohydrates, nucleic acids; δ(C-O-H) |
| 1059 | Carbohydrates, glycoproteins; δ(C-O-H) |
| 1084 | Nucleic acids; ν_s_(PO_2_^-^)  Phospholipids; ν_s_(PO_2_^-^) |
| 1122 | Ribose (RNA); ν(C-O) |
| 1156 | Poly/sugars; ν(CC-OC) |
| 1169 | Cholesterol esters; ν_as_(CO-O-C) |
| 1200 | Proteins (amide III); |
| 1236 | Nucleic acids; ν_as_(PO_2_^-^)  Phospholipids; ν_as_(PO_2_^-^) |
| 1285 | Nucleic acids; ν_as_(PO_2_^-^)  Phospholipids; ν_as_(PO_2_^-^) |
| 1315 | Phospholipids; ν(CH_2_) |
| 1338 | Phospholipids, fatty acids, triacylglycerols; ν(CH_2_) |
| 1368 | Proteins, amino acids; δ(CH_2_) |
| 1384 | Proteins; δ(CH_3_) in aliphatic chains |
| 1396 | Free amino acids; ν_s_(COO^-^) |
| 1421 | Fatty acids; δ(CH_2_) in the α-CH_2_ groups |
| 1443 | Proteins; δ(CH_2_, CH_3_) |
| 1457 | Proteins; δ(CH_2_, CH_3_) |
| 1494 | Proteins (amide II); δ(N-H) and ν(C-N) |
| 1514 | Tyr (proteins); ν(CC) of the ring |
| 1541 | Proteins (amide II); δ(N-H) and ν(C-N) |
| 1559 | Proteins (amide II); δ(N-H) and ν(C-N) |
| 1625 | Intercellular aggregations of cellular proteins |
| 1632 | β-sheets in proteins n(amide I); ν(C=O) and δ(N-H) |
| 1651 | α-helices in proteins (amide I); ν(C=O) and δ(N-H) |
| 1680 | β-turns in proteins (amide I); ν(C=O) and δ(N-H) |
| 1699 | Nitrogenous bases of nucleic acids; ν(C=O) |
| 1720 | Fatty acids; ν_acid_(C=O) |
| 1744 | Triacylglycerols; ν_ester_(C=O) |
| 2852 | Long chain FAs; ν_s_(CH_2_) |
| 2874 | Proteins, lipids, nucleic acids; ν_s_(CH_3_) |
| 2924 | Lipids and proteins; ν_as_(CH_2_) |
| 2960 | Lipids and proteins; ν_as_(CH_3_) |
| 3011 | Unsaturated lipids; ν_s_(=CH) |

ν – stretching mode, as – asymmetric, s – symmetric; δ – in-plane deformations; Tyr – tyrosine, FAs- fatty acids.

**Table S2.** RS band positions and their assignments to vibrational modes of biomolecules (10–17).

| *Band position [cm^-1^]* | *Assignment to biomolecules and vibrational modes* |
| --- | --- |
| 724 | A (nucleic acids); ring breathing  Phospholipids; ν_s_(N^+^(CH_3_)_3_) of choline group |
| 756 | Cyt. c and c_1_; ν(porphyrin ring) |
| 790 | Nucleic acids; ν_s_(PO_2_) |
| 827 | Tyr (proteins); ν_s_(C-C-N^+^) |
| 855 | Tyr (proteins); ν_s_(C-C-N^+^) |
| 886 | Proteins; δ(CH_2_) |
| 902 | Trp (proteins); ν(C-C) and ν(C-N) |
| 925 | Cholesterol esters (lipid droplets); ν(C-C) |
| 937 | Proteins; ν(C-C_α_) |
| 964 | Carbohydrates; ν(C-O) |
| 1007 | Phe (proteins); ring breathing |
| 1038 | Cross-linked Phe (proteins) |
| 1085 | Fatty acids; ν(C-C) |
| 1095 | Phosphate-containing molecules; ν_s_(PO_2_) |
| 1130 | Phospholipids; ν(C-C)  *Cyt. c and c_1_; ν(porphyrin ring) |
| 1178 | Tyr, Phe (proteins); δ(C-H) |
| 1211 | Tyr, Phe, Trp, Hyp (proteins); τ(CH_2_) |
| 1253 | Proteins (amide III); ν(C-N), δ(N-H), ν(CH_3_-C) |
| 1267 | Unsaturated lipids; δ(=CH) |
| 1306 | Unsaturated fatty acids; τ(CH_2_/CH_3_) |
| 1311 | G (nucleic acids); ring breathing, δ(C-H)  *Cyt. c and c_1_; δ(C-H) |
| 1340 | Nucleic acids, proteins; ring breathing; δ(C-H)  Lipids; τ/δ(CH_2_/CH_3_) |
| 1445 | Proteins, lipids; δ(CH_2_, CH_3_) |
| 1449 | Proteins, lipids; δ(CH_2_, CH_3_) |
| 1587 | A, G (nucleic acids); ring breathing  *Reduced cyt. c, c_1_ and b; ν(methine bridges – C_a_C_m­_,C_a_C_m_H bonds) |
| 1660 | Unsaturated fatty acids; ν(C=C) |
| 2853 | Long chain fatty acids; ν_s_(CH_2_) |
| 2886 | Proteins; ν(C-H)-CH_2_ |
| 2895 | Lipids; ν_s_(-C-H)-CH_3_ |
| 2933 | Proteins; ν_as_(CH_3_) |
| 3014 | Unsaturated fatty acids; ν(=C-H) |

ν – stretching mode, as – asymmetric, s – symmetric; δ – in-plane deformations; γ - out-of-plane deformations; τ – twisting; cyt – cytochromes; A – adenine; C – cytosine; G – guanine; U – uracil; T – thymine; Hyp – proline; Tyr – tyrosine; Phe – phenylalanine; Trp - tryptophan; ^*^Bands at 1130, 1311, and 1587 cm^-1^ are assigned to cytochromes only if the ~750 cm^-1^ band is present.

**Table S3.** A number of FTIR and RS vibrational spectra destined for further analysis.

|  | *Number of acquired spectra* | | | |
| --- | --- | --- | --- | --- |
| *Technique* | ***FTIR*** | ***RS*** | | |
| Phase | - | proteinaceous | lipidic | % lipidic |
| 6h | 34 | 439 | 43 | **9.1** |
| 6h NC | 30 | 465 | 13 | 2.7 |
| 2d | 35 | 447 | 30 | **6.3** |
| 2d NC | 30 | 440 | 22 | 4.8 |
| 7d | 33 | 270 | 37 | **12.0** |
| 7d NC | 30 | 419 | 41 | 8.9 |
| 14d | 31 | 329 | 47 | **12.3** |
| 14d NC | 30 | 462 | 38 | 7.6 |


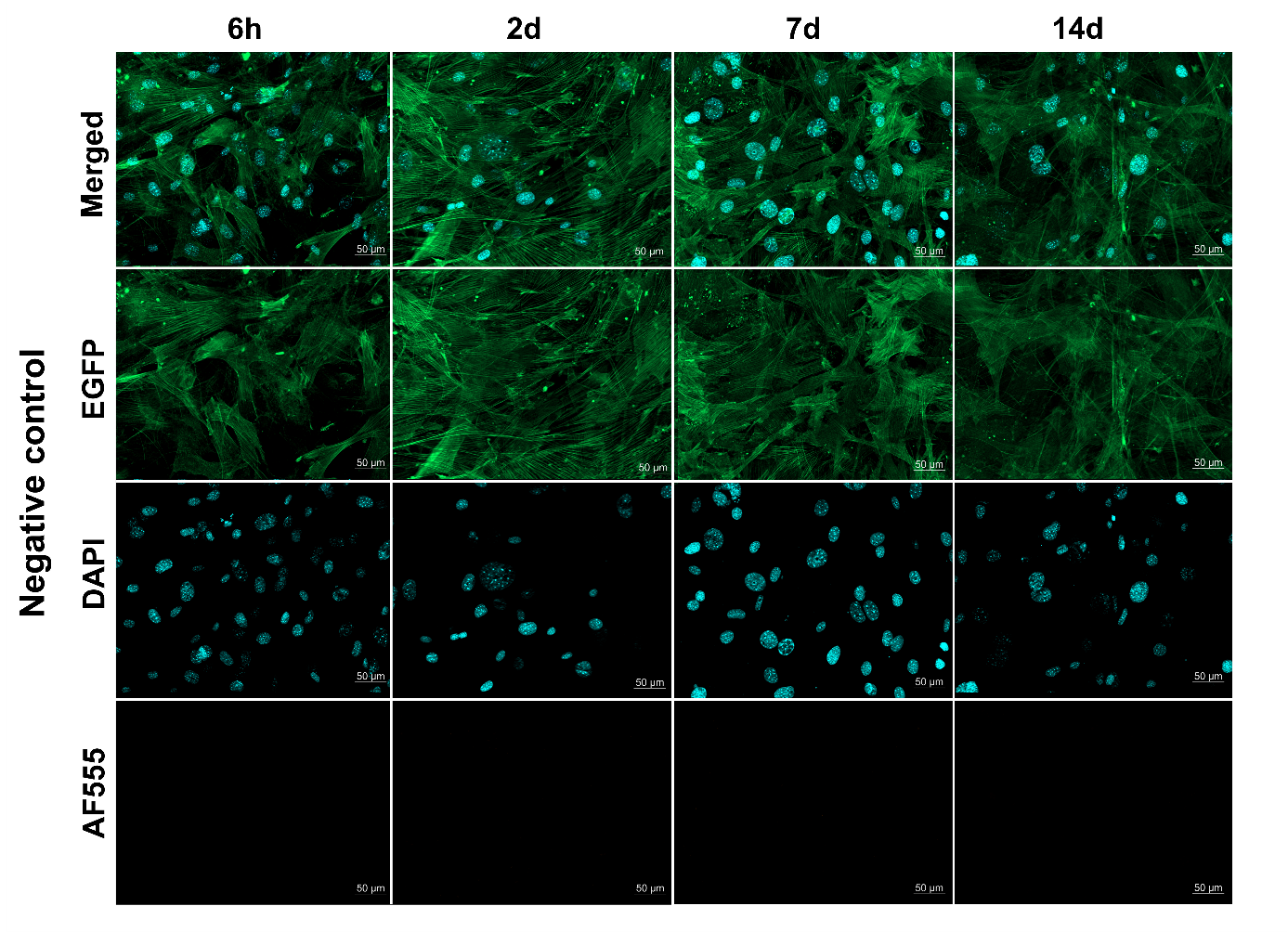


**Figure S1** AD-MSCs control cell culture. Immunofluorescence images: EGFP (green) – actin cytoskeleton; DAPI (blue) – nuclei; AF555 (orange) – lipids (magnification 200×, scale bar = 50 µm).


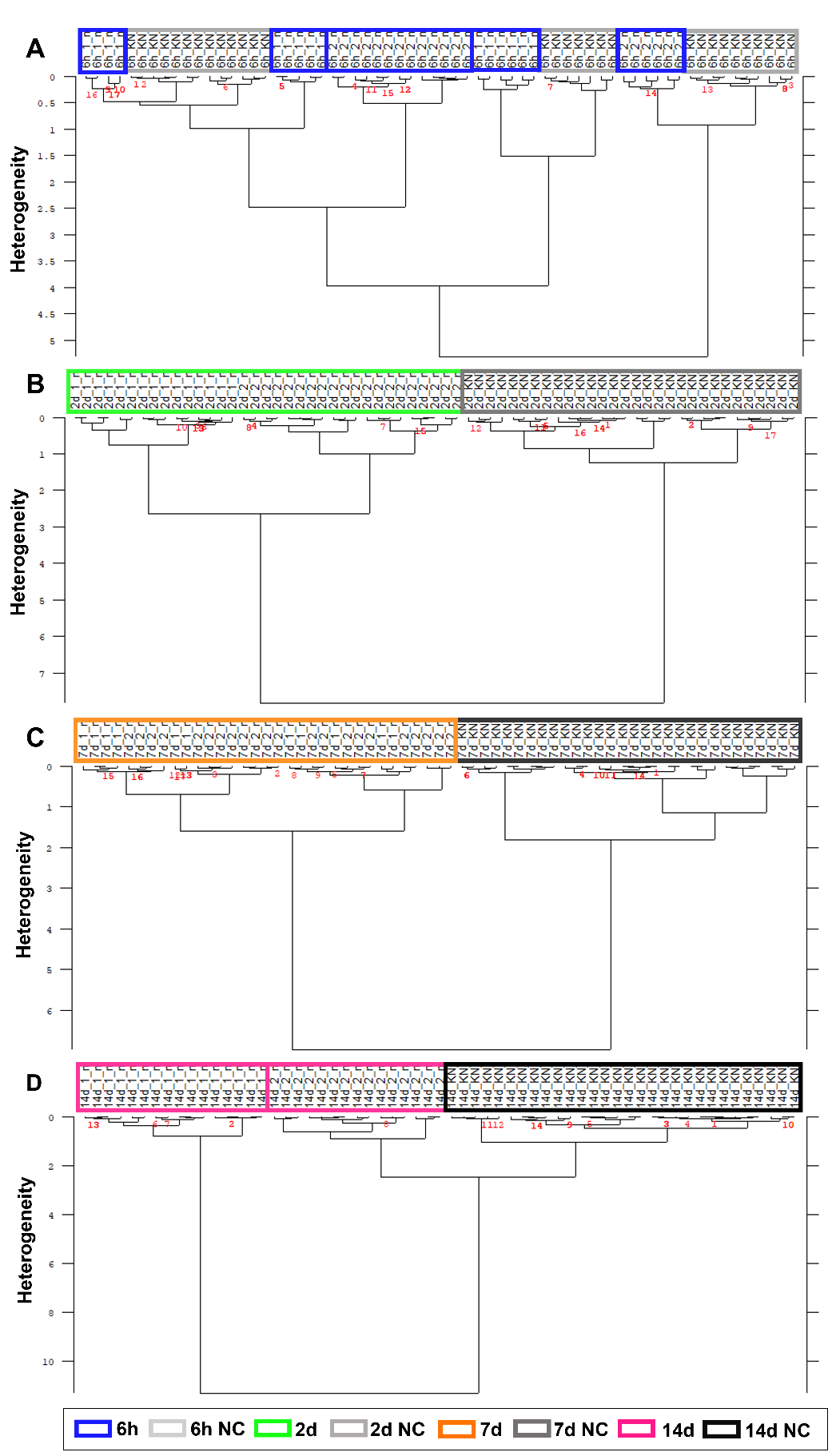


**Figure S2** HCA dendrograms calculated for the second derivative FTIR spectra in the regions of 3050-2800 cm^-1^ and 1750-920 cm^-1^. Clustering of the adipogenesis phases at selected time points after its induction **(A)** 6 h; **(B)** 2 days; **(C)** 7 days; **(D)** 14 days, with the corresponding negative controls (NC).


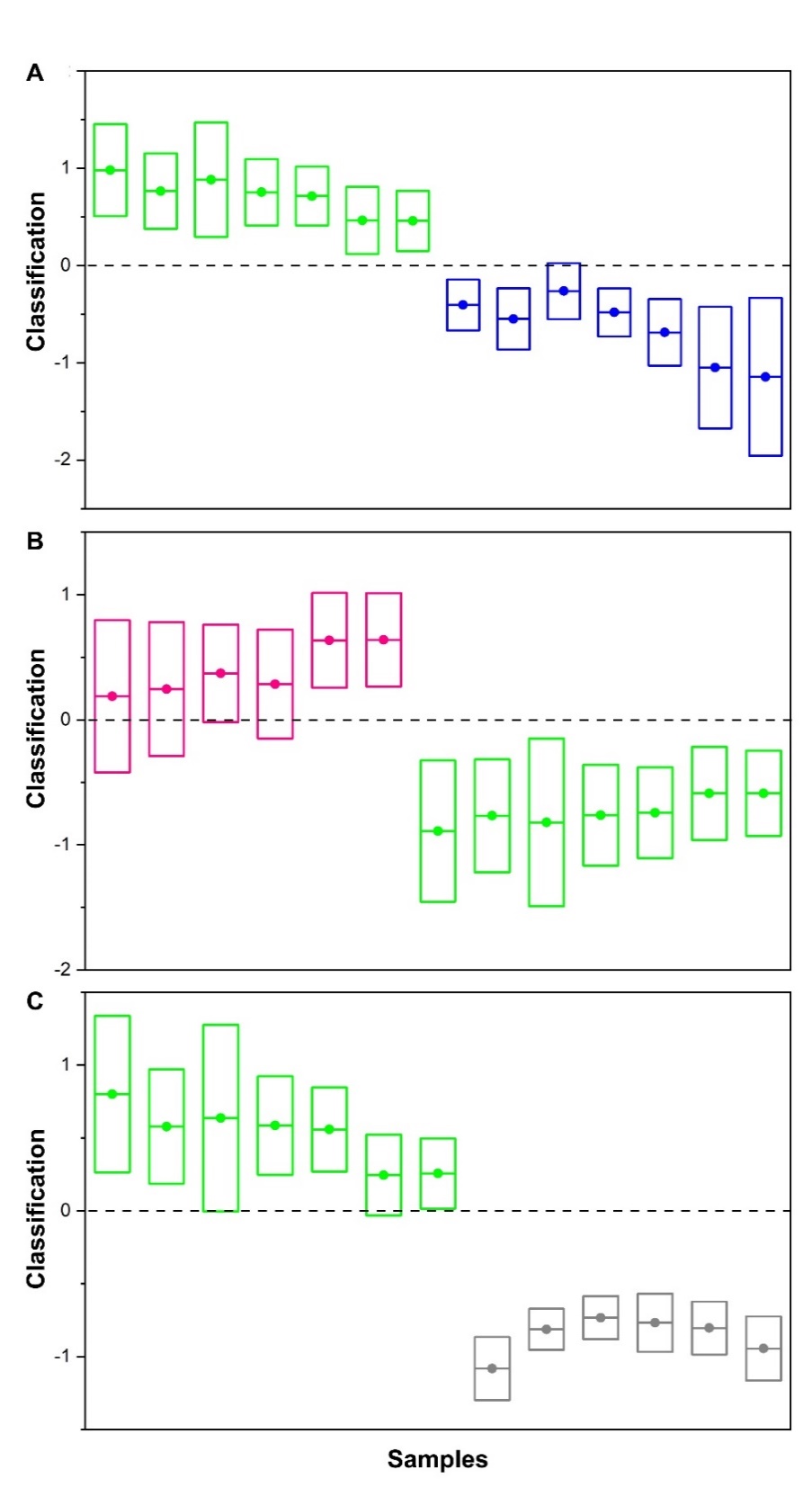


**Figure S3** Results of PLSR classification for models constructed based on FTIR spectra of the cells cultured in the first biological replicate. **(A)** 6h vs. early phase (2d), **(B)** early phase vs. late phase (14d), **(C)** early phase vs. its negative control. Correct classification was assigned to predicted values > +0.25 and < −0,25; spectra with the predicted value in the range of −0,25 ÷ +0.25 were considered unclassified. The boxes represent the standard deviation.


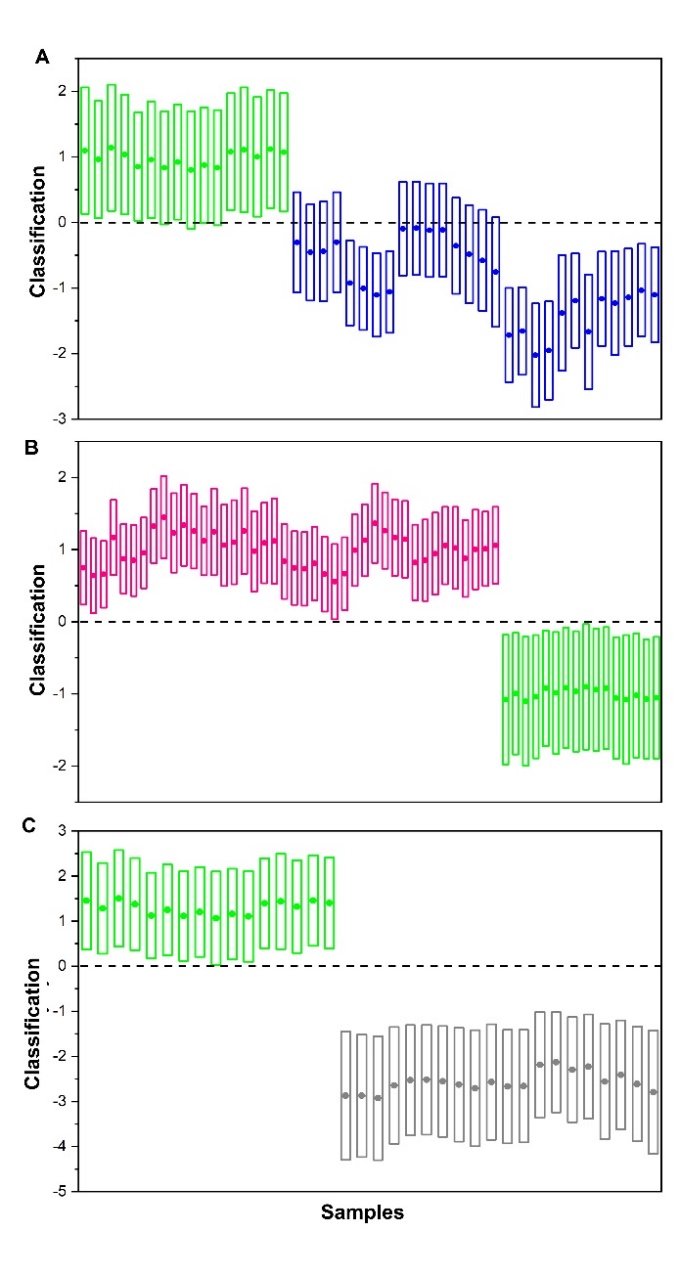


**Figure S4** FTIR-based PLSR prediction results of the MSCs transformation prepared in the second biological replicate. **(A)** 6h vs. early phase (2d), **(B)** early phase vs. late phase (14d), **(C)** early phase vs. its negative control. Correct classification was assigned to predicted values > +0.25 and < −0,25; spectra with the predicted value in the range of −0,25 ÷ +0.25 were considered unclassified. The boxes represent the standard deviation.

**References:**

1. Sahu, R. K., S. Argov, A. Salman, M. Huleihel, N. Grossman, Z. Hammody, J. Kapelushnik, and S. Mordechai. 2004. Characteristic absorbance of nucleic acids in the Mid-IR region as possible common biomarkers for diagnosis of malignancy. *Technol. Cancer Res. Treat.* **3**: 629–638

2. Szafraniec, E., E. Wiercigroch, K. Czamara, K. Majzner, E. Staniszewska-Slezak, K. M. Marzec, K. Malek, A. Kaczor, and M. Baranska. 2018. Diversity among endothelial cell lines revealed by Raman and Fourier-transform infrared spectroscopic imaging. *Analyst*. **143**: 4323–4334.

3. Perez-Guaita, D., K. Kochan, M. Martin, D. W. Andrew, P. Heraud, J. S. Richards, and B. R. Wood. 2017. Multimodal vibrational imaging of cells. *Vib. Spectrosc.* **91**: 46–58. [online] http://dx.doi.org/10.1016/j.vibspec.2016.07.017.

4. Wiercigroch, E., E. Staniszewska-Slezak, K. Szkaradek, T. Wojcik, Y. Ozaki, M. Baranska, and K. Malek. 2018. FT-IR Spectroscopic Imaging of Endothelial Cells Response to Tumor Necrosis Factor-α: To Follow Markers of Inflammation Using Standard and High-Magnification Resolution. *Anal. Chem.* **90**: 3727–3736.

5. Banyay, M., M. Sarkar, and A. Gräslund. 2003. A library of IR bands of nucleic acids in solution. *Biophys. Chem.* **104**: 477–488.

6. Whelan, D. R., K. R. Bambery, P. Heraud, M. J. Tobin, M. Diem, D. McNaughton, and B. R. Wood. 2011. Monitoring the reversible B to A-like transition of DNA in eukaryotic cells using Fourier transform infrared spectroscopy. *Nucleic Acids Res.* **39**: 5439–5448.

7. Staniszewska, E., K. Malek, and M. Baranska. 2014. Rapid approach to analyze biochemical variation in rat organs by ATR FTIR spectroscopy. *Spectrochim. Acta - Part A Mol. Biomol. Spectrosc.* **118**: 981–986.

8. Molony, C., J. McIntyre, A. Maguire, R. Hakimjavadi, D. Burtenshaw, G. Casey, M. Di Luca, B. Hennelly, H. J. Byrne, and P. A. Cahill. 2018. Label-free discrimination analysis of de-differentiated vascular smooth muscle cells, mesenchymal stem cells and their vascular and osteogenic progeny using vibrational spectroscopy. *Biochim. Biophys. Acta - Mol. Cell Res.* **1865**: 343–353. [online] http://dx.doi.org/10.1016/j.bbamcr.2017.11.006.

9. Lewis, R. N. A. H., and R. N. McElhaney. 2013. Membrane lipid phase transitions and phase organization studied by Fourier transform infrared spectroscopy. *Biochim. Biophys. Acta - Biomembr.* **1828**: 2347–2358. [online] http://dx.doi.org/10.1016/j.bbamem.2012.10.018.

10. Bik, E., A. Dorosz, L. Mateuszuk, M. Baranska, and K. Majzner. 2020. Fixed versus live endothelial cells: The effect of glutaraldehyde fixation manifested by characteristic bands on the Raman spectra of cells. *Spectrochim. Acta - Part A Mol. Biomol. Spectrosc.* **240**: 118460. [online] https://doi.org/10.1016/j.saa.2020.118460.

11. Majzner, K., S. Chlopicki, and M. Baranska. 2016. Lipid droplets formation in human endothelial cells in response to polyunsaturated fatty acids and 1-methyl-nicotinamide (MNA); confocal Raman imaging and fluorescence microscopy studies. *J. Biophotonics*. **9**: 396–405.

12. Czamara, K., K. Majzner, M. Z. Pacia, K. Kochan, A. Kaczor, and M. Baranska. 2015. Raman spectroscopy of lipids: A review. *J. Raman Spectrosc.* **46**: 4–20.

13. Prescott, B., W. Steinmetz, and G. J. Thomas. 1984. Characterization of DNA structures by laser Raman spectroscopy. *Biopolymers*. **23**: 235–256.

14. Wu, M., K. Pu, T. Jiang, Q. Zhai, Z. Ma, H. Ma, F. Xu, Z. Zhang, and Q. Wang. 2021. Early label-free analysis of mitochondrial redox states by Raman spectroscopy predicts septic outcomes. *J. Adv. Res.* **28**: 209–219. [online] https://doi.org/10.1016/j.jare.2020.06.027.

15. Bik, E., N. Mielniczek, M. Jarosz, J. Denbigh, R. Budzynska, M. Baranska, and K. Majzner. 2019. Tunicamycin induced endoplasmic reticulum changes in endothelial cells investigated: In vitro by confocal Raman imaging. *Analyst*. **144**: 6561–6569.

16. Matthäus, C., T. Chernenko, J. A. Newmark, C. M. Warner, and M. Diem. 2007. Label-free detection of mitochondrial distribution in cells by nonresonant Raman microspectroscopy. *Biophys. J.* **93**: 668–673.

17. Meade, A. D., C. Clarke, F. Draux, G. D. Sockalingum, M. Manfait, F. M. Lyng, and H. J. Byrne. 2010. Studies of chemical fixation effects in human cell lines using Raman microspectroscopy. *Anal. Bioanal. Chem.* **396**: 1781–1791.
